# Development and utility of a close contact information management system for the COVID-19 pandemic

**DOI:** 10.1186/s12889-021-12355-7

**Published:** 2021-12-11

**Authors:** Jiali Long, Rong He, Shen Tian, Yefei Luo, Mengmeng Ma, Wen Wang, Yuehong Wei, Jun Yuan

**Affiliations:** 1 Guangzhou Center for Disease Control and Prevention, Guangzhou, 510440 Guangdong China; 2 School of Public Health, Guangzhou Medical University, Guangzhou, 511436 Guangdong China

**Keywords:** The COVID-19 close contact information management system, The COVID-19 pandemic, Disease control

## Abstract

**Background:**

Since the outbreak started in 2019, COVID-19 pandemic has a significant global impact. Due to the highly infective nature of SARS-CoV-2, the COVID-19 close contacts are at significant risk of contracting COVID-19. China’s experience in successfully controlling COVID-19 emphasized the importance of managing close contacts because this strategy helps to limit potential infection sources, prevent the unconscious spread of COVID-19 and thus control this pandemic. As a result, to understand and consider the management of close contacts may be beneficial to other countries. However, managing close contacts is challenging owing to the huge number of close contacts and a lack of appropriate management tools and literature references.

**Methods:**

A new system called the COVID-19 Close Contact Information Management System was developed. Here we introduced the design, use, improvement and achievements of this system.

**Results:**

This system was designed from the standpoint of the Centers for Disease Control and Prevention in charge of managing close contacts. Two main functions and eight modules/themes were ultimately formed after two development stages. The system introduces what information need to be collected in the close contact management. Since the system allows information flow across cities, the geographical distance and administrative regional boundaries are no longer obstacles for managing close contacts, which promotes the management of each close contact. Moreover, when this system is used in conjunction with other data tools, it provides data assistance for understanding the COVID-19 characteristics and formulating targeted COVID-19 control policies. To date, the system has been widely used in Guangdong Province for over 1 year and has recorded tens of thousands of pieces of data. There is sufficient practical experience to suggest that the system is capable of meeting the professional work requirements for close contact management.

**Conclusions:**

This system provides a new way to manage close contacts and restrict the spread of COVID-19 by combining information technology with disease prevention and control strategies in the realm of public health. We hope that this system will serve as an example and guide for those anticipating similar work in other countries in response to current and future public health incidents.

## Background

Coronavirus disease 2019 (COVID-19) is an emerging contagious disease caused by a new coronavirus called Severe Acute Respiratory Syndrome Coronavirus 2 (SARS-CoV-2) [1]. This emerging disease is characterized by rapid spread, atypical clinical symptoms and high contagiousness [2]. COVID-19 spreads via respiratory droplets or close contact, with an estimated basic reproductive number (R0; defined as the average number of secondary cases attributable to infection by an index case after this case is introduced into a susceptible population) of up to 5.7 [2]. People at any age are susceptible and can become seriously ill or die [1]. This contagious nature caused a surge of COVID-19 patients, and the tremendous influx of patients further contributed to the overwhelming hospitalizations, inadequate healthcare workforce, and the depletion of drugs and medical supplies, thereby exacerbating the uncontrollable and devastating COVID-19 situation [3]. It is worth mentioning that many countries have reported these similar challenges and emphasized the obviously negative impact of these challenges in their fight against the COVID-19 [3–5]. On March 11, 2020, the World Health Organization (WHO) declared that the COVID-19 outbreak constituted a global pandemic [6]. As of 21 October, 2021, over 239 million confirmed cases have been reported worldwide, with 4.9 million people dying [1]. China is one of the first countries to face these challenges but control this epidemic. Even though mainland China has already reported 96,665 confirmed COVID-19 cases, most of cases have recovered with only 518 active cases as of October 21, 2021 [7]. This amazing achievement attributed to China’s successful COVID-19 response such as the implementation of strict lockdown, active case surveillance, rapid case diagnosis, effective close contact management to reduce the source of infection, the application of modern technology, increased government support to solve the shortage of healthcare workforce and medical supplies, and the development of vaccines to protect susceptible populations, which may serve as a guide for other countries in their fight against the COVID-19 pandemic. China especially stressed the importance and necessity of managing close contacts. Close contacts in China was defined as those who have close contact with suspected, confirmed or diagnosed cases 2 days before illness onset, or with asymptomatic carriers 2 days before sampling but have not taken effective protection [8]. Due to the highly infectious nature of COVID-19, close contacts are regarded to be at high risk of contracting COVID-19. Therefore, close contact management is a type of active case surveillance that aims at control potential infection sources. China advocated for all close contacts to be quarantined or medically observed for 14 days, which have been written in the COVID-19 Prevention and Control Guidelines [8] and implemented. Admittedly, the management of close contacts, such as information registration, personnel management and data statistics is challenging. The number of close contacts always represent geometric multiples of the number of confirmed cases [7]. However, regular electronic tables or paper records are not adequate to meet the work requirements that involved managing large numbers of people. More severely, the tools available to assist in the close contact management in a systematical, intelligent, and cross-geographical manner are extremely limited [9]. Meanwhile, many researchers still focus on the hospital management or disease assessment and diagnosis of the confirmed cases [10–14], and the literature or reports on the systematic management of close contacts are almost non-existent, implying that the management of close contacts lacks references. As a result, to urgently construct a useful tool suitable for managing a huge number of close contacts during times in COVID-19 pandemic is a challenging task [15].

In this regard, we would like to introduce the COVID-19 Close Contact Information Management System (“close contact system”), which combines information technology with public health management. It was developed and utilized by Guangdong Province, China, and was demonstrated to be effective against the COVID-19 pandemic. This system collects and sorts the information of close contacts, achieves cross-regional information flow and feedback in Guangdong Province, and finally promotes the management of all close contacts across the province and makes remarkable contributions to the control of COVID-19. In addition, the system also provides a platform for data support for policy designations and optimizations. This paper may help to address a research gap in the field of close contact management. We describe the design, use, improvement and achievements of the system, and hopes the experiences shared here will serve as inspiration and guidance for those participating similar work in other countries to develop a tailored approach to create a platform in response to infectious diseases that occur now or in the future.

## Methods

### Design

The development of the close contact system was divided into two stages: system design, and multiple training and practice optimization.

### Participants

The main participants in the first stage were the professionals from the lead unit for system development, namely the Guangdong Center for Disease Control and Prevention (hereinafter referred to as “Guangdong CDC”), and system development programmers.

The main participants in the second stage were the planned users of the system, namely the staff of all CDCs at different levels in Guangdong Province. It is worth mentioning that Guangdong Province contains 21 cities, all of which are involved in the system’s use and promotion. Besides, the CDCs are one of the main units that are fighting the COVID-19 pandemic. They are often responsible for conducting epidemiological investigations of cases or close contacts, writing reports, assisting in the transfer of cases to hospitals, centrally quarantining and managing close contacts in designated hotels, and sanitizing the environment. Many CDC staff who use this system have direct contact experience with COVID-19 cases and/or in conducting epidemiological investigations of cases.

Non-CDC staff such as intern students did not participate in the system’s development, use and optimization to ensure professionalism.

### Procedure

At the beginning of the COVID-19 outbreak, Guangdong Province did not have a suitable tool for storing, managing, and analyzing the information of close contacts. Therefore, it was urgent to develop a tool suitable for close contact management in Guangdong Province.

#### Stage 1: system development

The primary goal of designing the close contact system was to collect as much information about close contacts as possible and then promote the management of all close contacts. This system was defined from the perspective of the CDCs responsible for managing close contacts and it referred to previous experience in managing other infectious diseases and advice from other experts. Under the pressure of the urgent needs and limited time but with a lack of references, professionals at the Guangdong CDC directly determined the system framework and information needed to be collected. These requirements and concepts were transformed into system items after fully communicating with the system development programmers, and the first version of the close contact system was then successfully constructed.

#### Stage 2: multiple training and practice optimization

After the system was developed, the Guangdong CDC provided initial training on the use of the system to all other CDCs in Guangdong Province via live video broadcasts. In the process of system practice and popularization, users from all CDCs were encouraged to provide comments and suggestions regarding the system, mainly with respect to the completeness of personal information, the practicality of functions, the clarity of logic of the system program, and the fluency and operational stability of the system. These comments and suggestions would be summarized and reviewed later by the Guangdong CDC, and the audited comments and suggestions would be handed over to the system programmers who continued to provide technical support at all times during the subsequent trial period, resulting in adjustments to system items and/or functions. Training regarding the adjustments will be provided in a timely manner by means of live video or PPT.

## Results

### Stage1: system design

At the beginning of system design, the close contact system was fully designed to provide accurate and complete recording of all close contact information while also ensuring information security.

The close contact system has two main functions. One function is information collection, which consists of five major themes. Table 1 shows the detailed items for these five themes. Furthermore, the system specifies the lengths and formats of information input. For example,

**Table 1:** The initially determined information framework for input and management

	System function	Main modules/themes	Concrete content	Remarks
Stage 1	1. Information collection	(1) Location	Management unit; The district/county where the unit is located	
		(2) Basic information of close contact	Number, Name; ID card; Gender; Age; Occupation; Residence address; Work unit and address; Telephone number	
		(3) Epidemiological information (mainly about the relationship to the case)	Name of case; ID card of case; Relationship to the case; The location of contact, The manner of contact, The means of transportation used at the time of contact; Frequency of contact, Date of last contact; Date to be discharged	
		(4) Management information of close contact	Quarantine or not; The method of quarantine; The date of quarantine; The location of quarantine	
		(5) Laboratory results and prognosis	Time of sampling, Test result, Onset or not, Symptoms, Prognosis	
	2. Information processing	(1) Basic module	Input (a single message), Modify, Delete (a single message), Query, Export	To reduce the likelihood of errors, the system restricts the permission of batch entry and batch deletion.
		(2) Information transfer module	Transfer in, Transfer out, Share	
		(3) Authority module	For example, the information turnover between two district-level CDCs in the same city needs to be reviewed and approved by the city-level CDC, and the information turnover between district-level CDCs in two cities needs to be reviewed and approved by both the city-level CDCs of the two cities and their common province-level CDC.	

multiple-choice questions are used to enter relatively fixed information such as “Frequency of contact”. Answers to open-ended questions, such as “Reasons for home isolation”, are encouraged to be detailed. The second function is information processing. The function is divided into three modules: basic module, information transfer module and authority module. First, the basic module includes functions such as “Input”, “Modify”, “Delete”, “Query”, and others. Second, the information transfer module allows information to flow across cities, removing the geographical distance and administrative regional boundaries as obstacles to managing close contacts. The system information can be circulated across different units at the same level or at different levels in the province with the “Transfer in”/“ Transfer out” function. This function also assists the unit responsible for a specific close contact to be aware of the existence of this individual so that the unit can contact and manage this person. Another scenario is a case and the case’s close contacts are located in different locations and thus managed by different units. When the unit in charge of the COVID-19 case management wants to learn more about the case’s close contacts, it can use the “Share” function to access the information for close contact stored in the system after the application is approved. The difference between the two function is that the “Share” function only allow users to read information, whereas the “Transfer in”/” Transfer out” function allow users to transfer ownership such as modification. Third, the authority module performs level-by-level reviews of some critical operations to reduce the likelihood of errors, such as reviewing the operation that deletes data in batches. Similarly, the information of close contacts can only be transmitted across different units with the approval of a higher-level CDC. This authority module achieves directly vertical management from senior to junior units and feedback from junior to senior units.

Furthermore, nonspecific accounts are not permitted to login to the system to view or modify personal information, which fully ensures personal information security.

### Stage 2: multiple training and practice optimization

Practice has indicated that the initial system design (Table 1) basically met the management needs for close contacts, which included recording accurate and detailed information regarding close contacts and providing a platform for all CDCs in the province to exchange this information. The further optimization of this system has frequently been linked to the progression of the pandemic and changes in regional policies [8, 16], and mainly reflected the adjustments to system items and functions (Table 2). Moreover, because some content was noticed to be easily mistyped during the information input process, the system has implemented logical constraints. For example, if the data was uploaded to the system on June 1, the “Input time” defaulted to the current day, and other dates (such as June 2) were not allowed to be selected.

**Table 2:** Adjustments of system items and modules

	System function	Modified module	Content of the modification	Reason
Stage 2	1. Information collection	(1) Location	Add: Have issued an administrative document applying assistance in inquiring information about close contacts?; The planned recipient of the document	Sometimes close contacts are not in Guangdong Province but have gone to other provinces or abroad.
		(3) Epidemiological information (mainly about the relationship to the case)	Add: Whether he/she is a close contact of another close contact(A); The name and ID card of that close contact(A); Whether that close contact(A) converted to a case and the date; Enter from abroad or not, Enter time, Flight number; Seat number	On the one hand, the number of local cases in Guangdong Province remains zero, while the proportion of imported cases from other countries has soared. On the other hand, the updating of national guidelines and administrative documents has also led to changes in information collection. For example, the Disease Prevention And Control Bureau added definitions of “imported cases from abroad, close contacts of close contacts” in the seventh edition of the COVID-19 Prevention and Control Guidelines [7], requiring CDC staff to also pay attention to the risk of infection in these groups.
	2. Information processing	(1) Basic module	Add: Batch input; Batch delete	The number of new close contacts in one day has soared. For example, the number of new close contacts in one day in Guangzhou could be as high as 503. Inputting information one by one no longer met the needs of work. In addition, users were already familiar with the system after months of practice. Therefore, the Guangdong CDC took Guangzhou as a pilot city and made the batch input function module available to Guangzhou users. Meanwhile, the system programmers provided technical support at any time, hoping to complete the pilot work as soon as possible, and then steadily promote this function throughout Guangdong Province.
		(3) Authority module	Add:Auditing of deleted information (For example, the information can only be deleted after the application of the subordinate unit is reviewed and approved by the superior unit, otherwise the information cannot be delete.)	The level-by-level auditing of the “Delete” function not only strengthens the “District-City-Province” vertical hierarchical management but also avoids the error of data being deleted by mistake.

The first large-scale live video training provided CDC staff with a preliminary understanding of the system, but this training was insufficient. Relevant explanations for the above-mentioned system optimizations in stage 2 were transmitted to the CDCs in a timely manner by live video or PPT, which served as follow-up training for the system. The Guangdong CDC also formulated standards for some ambiguous problems encountered during the work process and then transmitted them to the other CDCs. These trainings are temporary and brief, but they are timely and significant in ensuring that all CDCs can process information uniformly and thus maintain normal system operation.

#### Application of the close contact system

This is the first close contact system which has been fully used and promoted in a whole province, addressing the deficiency in this area.

Following its inception, the system was promoted throughout 21 cities in Guangdong Province. In February 2020, the first piece of data for a close contact was entered. By October 21, 2021, the system had been in use for over 600 days. Taking Guangzhou, a city in Guangdong Province, as an example, the system has received data for as many as 55,888 close contacts identified in Guangzhou, of which 97.62% have completed or are undergoing 14-day quarantines in Guangzhou, and 2.37% are not quarantined (for example, someone has moved to another province). Only 7 close contacts (0.01%) have yet to be contacted and managed due to incorrect information, and this situation rarely happened in the later period with the assistance of the police. Therefore, it is clear that close contacts are managed as they should be with the assistance of the system.

**Fig. 1:**
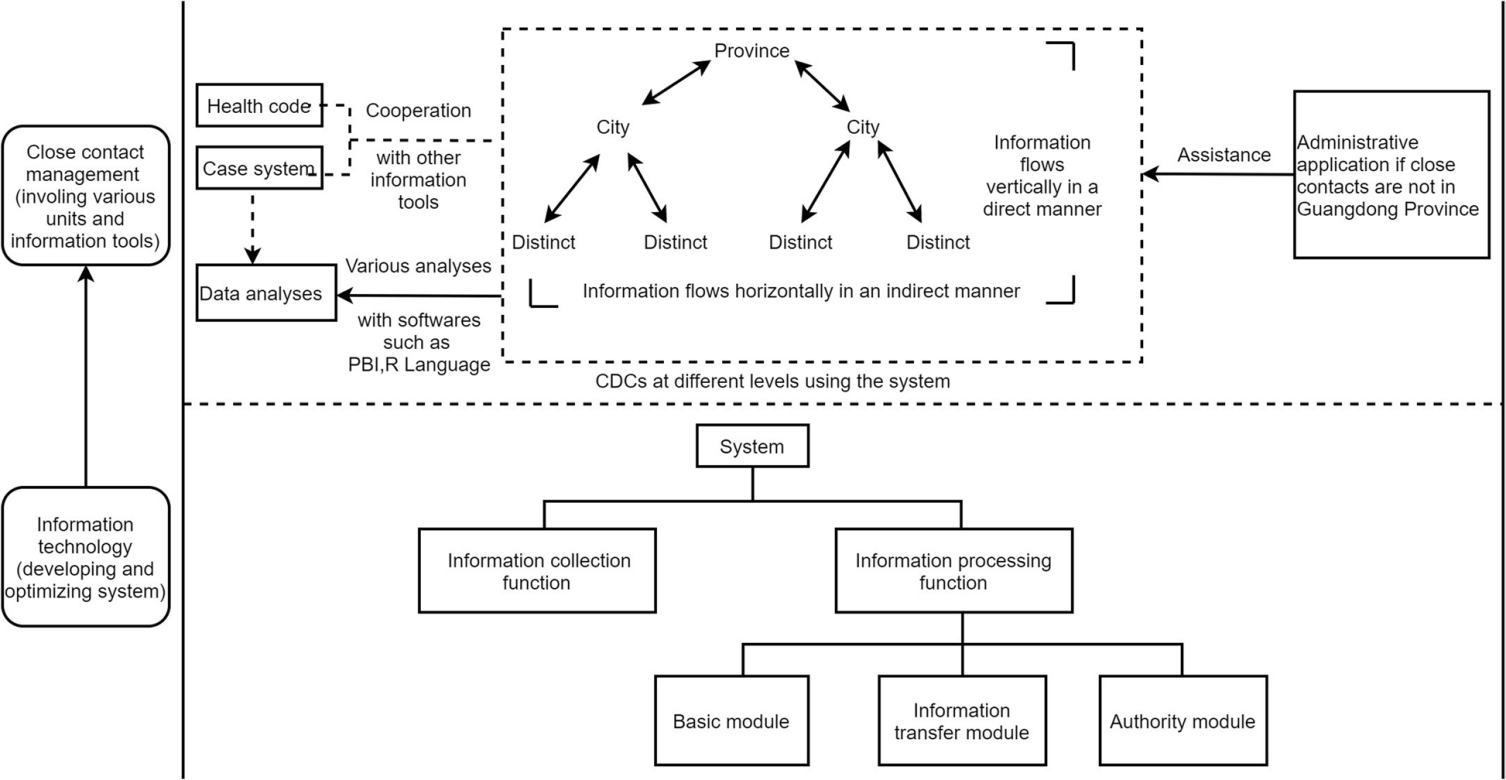
The framework and application of close contact system in Guangdong Province. (1) Information technology provides powerful support for the construction and optimization of the system. The system framework is shown below the long dotted line in Fig. 1. The system contains two main functions that can be optimized at any time based on work requirements. (2) As the system is gradually improved and optimized, it plays an increasingly significant role in the management of close contacts. As shown in the dashed box, “District-City-Province” directly vertical information communication and “Distrct-Distrct” / “City-City” indirectly horizontal information communication in Guangdong province have been achieved in the systems which were used by CDCs at different levels. Besides, the extended application of the system is shown outside the dashed box. Cooperation with other information tools, such as the health code and the case system, helps to perform data analyses and thus helps to understand the characteristics of COVID-19 and conduct disease control. Furthermore, the information of close contacts outside Guangdong province is also managed with the assistance of administrative applications, expanding the effective management scope of the system

The next step in the data collection process is to make full use of the system’s close contact data. Detailed and complete system data directly help to understand the characteristics of COVID-19. Meanwhile, the use of this system in conjunction with other information tools for analyzing the data has exhibited an enhanced effect on COVID-19 control. On the one hand, the system helps to control the flow of people through data association with the “Health code” to isolate potential infection sources as much as possible, cut off transmission routes of the virus, and protect susceptible population (Fig. 1). “Health code” is a special quick response code that was developed by China during the COVID-19 pandemic to categorize the risk of individuals contracting COVID-19. The health code has three colors that reflect different infection risks and correspond to different management policies: “Green code” means that the risk of personal infection is low, and people with the “Green code” can freely move between different cities in China; “Yellow code” means that people have a certain risk of infection, and they can only move conditionally; and “Red code” means that the person is at a very high risk of infection or has been diagnosed, such as being a close contact or a case, and this person must be quarantined or transferred to a designated hospital for treatment. In China, health code is widely used. The government requires people to show their personal health codes before entering public places like malls or taking public transportation. When a person is identified as a close contact and registered in the system, his or her health code will automatically change to “Red code”, which will restrict his or her movement. When the status of this close contact on the system is revised to “Released quarantine”, his or her health code will automatically change from “Red code” to “Green code” again. On the other hand, data from the close contact system and data from COVID-19 cases supplied by other systems are frequently analyzed jointly by staff using a variety of statistical software programs, such as Excel, Power BI (PBI), and R language (Fig. 1). On the basis of full data collection, the staff can quickly and flexibly conduct statistical analyses to provide data support for timely detection, rapid disposal, overall control, and continuous monitoring of COVID-19 control.

## Discussion

This paper introduced the design, use, improvement and achievements of the COVID-19 Close Contact Information Management System. This is the first widely used system for COVID-19 close contact management known to date. It combines information technology with disease control measures to investigate a new way to deal with the COVID-19 pandemic. The standardized information template of this system presents what information need to be collected in the management of close contacts, and assists staff in tracking the most recent status of close contacts. Since the system allows information to flow across cities, the geographical distance and administrative regional boundaries are no longer obstacles for managing close contacts. Even if these close contacts are scattered in different cities in Guangdong Province, the staff can promote the management of each close contact via system functions. These measures effectively manage close contacts who act as high-risk potential infection sources. Even if these close contacts contract COVID-19, the risk of the virus spreading will be significantly decreased, which will thereby reduce the risk of unrecognized COVID-19 transmission in the population. In addition, detailed and complete system data can also provide data support for understanding the characteristics of COVID-19 and formulating targeted policies to control this disease. In addition to the above advantages, the reliability of the system has also been demonstrated. To date, the system has been in wide use for near 2 year and has recorded tens of thousands of pieces of data. Its information security, operational stability, practicability, scalability and ease of maintenance have been demonstrated in practice. The system users consist of the professional staff from all CDCs in 21 cities in Guangdong Province. The number of users in Guangzhou alone is more than 90. In addition, no unexpected infections have occurred due to poor information management of close contacts. Therefore, there is sufficient practical experience to suggest that the system can meet the professional work requirements for close contact management in COVID-19 pandemic.

The world appears to be confronted with severe challenges on a regular basis. From the Nipah virus in 1998, to the severe acute respiratory syndrome(SARS) in 2003, to the H5N1 avian influenza in 2004, to the influenza A(H1N1) pandemic in 2009, and to recently COVID-19 in 2019 [17], emerging infectious diseases have broken every few years. These emerging diseases rapidly became disasters, placing enormous strain on many health systems and even causing social unrest. Previous experience suggests that countries will inevitably be affected when a disaster occurs. The COVID-19 pandemic confirms this view again, and the presence of mutant variants of SARS-CoV-2 has further exacerbated the situation of the ongoing wave of the COVID-19 pandemic due to their high transmissibility, reduced neutralization, and rapid global spread [18, 19]. As of 12 October, 2021, WHO declared that cases of Alpha variant have been reported from 195 countries, Beta variant from 145 countries, Gamma variant from 99 countries, and Delta variant from 191 countries [19]. To minimize the further spread of the COVID-19 epidemic, it is vital for governments to strengthen the regional sharing of successful experience in pandemic prevention and control. China’s experience in successfully controlling COVID-19 serves as a reminder that there is room for improvement when it comes to reducing pandemic risk. This paper introduces development and utility of the system, aiming to fill a research gap in the field of close contact management and provide a direct reference for others who are committed to infectious disease control. With adequate adaptations, and considering national differences and local aspects, elements of this system we have described might be helpful in guiding such efforts in response to current or future public health incidents. Besides, when confronted with the first generation of new viruses, the experience of urgently developing and optimizing a new system under a poor reference condition is worth promoting.

### Limitations and future scope of the work

The long-term management of close contacts also exposes some shortcomings of the system. Currently, the system is only popularized thorough Guangdong Province and it has not been introduced to the other provinces. When close contacts are located in another province, their information cannot be directly obtained or updated from the system. It is necessary to rely on administrative applications to obtain information regarding close contacts in other provinces. Besides, if the system is to be introduced to other provinces, the support from a higher authority is required. We also make mobility the next primary research point, with the hope of addressing the contradiction between mobility and privacy concerns to further promote this system. The current system only logs in to computer equipment. Once the risk of privacy breaches is reduced, this computer-only system may be able to be promoted to mobile devices, such as iPads or mobile phones.

## Conclusion

The development of this intelligent and convenient close connection system is a game-changing solution to effectively manage close contacts and thereby control the spread of COVID-19. When many countries encountered comparable challenges in their fight against COVID-19, it will be meaningful to sort out and share the successful anti-pandemic experience with other countries in need. We believe that the experience of this system may serve as inspiration and guidance for those anticipating similar work in other countries in response to current and future public health incidents.

## Data Availability

The datasets used and/or analyzed during the current study are available from the corresponding author on reasonable request.
